# Strategies for the development of moral resilience: voices of oncology nurses

**DOI:** 10.1590/0034-7167-2024-0210

**Published:** 2025-03-14

**Authors:** Mariane da Silva Barbosa, Ariel Siqueira Lemos, Liliane Alves Pereira, Taís Carpes Lanes, Flávia de Mello Disconsi, Kellen da Costa Pompeu, Jordana Lopes Carvalho, Graziele de Lima Dalmolin

**Affiliations:** I Universidade Federal de Santa Maria. Santa Maria, Rio Grande do Sul, Brazil; II Universidade Federal de Santa Maria, Hospital Universitário. Santa Maria, Rio Grande do Sul, Brazil; III Universidade Federal do Rio Grande. Rio Grande, Rio Grande do Sul, Brazil; IV Centro Universitário Franciscano. Santa Maria, Rio Grande do Sul, Brazil

**Keywords:** Medical Oncology, Psychological Resilience, Nursing, Ethics, Moral, Oncología Médica, Resiliencia Psicológica, Enfermería, Ética, Morale

## Abstract

**Objective:**

To describe the strategies used by nurses in a hemato-oncology service for the development of moral resilience.

**Methods:**

A qualitative, exploratory-descriptive study was conducted with nurses from the hemato-oncology department of a public hospital in Rio Grande do Sul, Brazil. Data collection occurred from January to May 2023 through semi-structured interviews. Content analysis was employed.

**Results:**

Fourteen nurses participated in the study. The issues raised regarding moral resilience resulted in two categories. The first focused on personal/self-awareness strategies, and the second on care strategies/patient care.

**Conclusion:**

It was observed that for hemato-oncology nurses, developing strategies for moral resilience is related to what these professionals consider relevant, not only for patient care but also for themselves.

## RESUMEN

**Objetivo:**

Describir las estrategias utilizadas por los enfermeros de un servicio de hemato-oncología para el desarrollo de la resiliencia moral.

**Métodos:**

Investigación cualitativa, exploratoria-descriptiva, realizada con enfermeros del servicio de hemato-oncología de una institución hospitalaria pública en Rio Grande do Sul, Brasil. La recolección de datos tuvo lugar entre enero y mayo de 2023, mediante entrevistas semiestructuradas. Se empleó el análisis de contenido.

**Resultados:**

Participaron en el estudio 14 enfermeros. Las cuestiones planteadas sobre la resiliencia moral resultaron en dos categorías. La primera se centró en estrategias a nivel personal/autoconocimiento y la segunda en estrategias de cuidado/cuidado del paciente.

**Conclusiones:**

Se observó que para los enfermeros de hemato-oncología, desarrollar estrategias de resiliencia moral está relacionado con lo que estos profesionales consideran relevante, no solo para el cuidado del paciente, sino también para ellos mismos.

## INTRODUCTION

With population aging and the rise of non-communicable chronic diseases, including cancer, healthcare professionals are increasingly required to possess technical and scientific preparation to provide qualified and safe care to these patients. A cancer diagnosis is often associated with death, making it difficult for the professionals involved in patient care to address and cope with this context^(1)^.

In this context, it becomes imperative for these professionals to have the means to cope with stress, burnout, fatigue, and moral distress, as in many instances, the patient’s illness goes beyond routine care or involves values that may not align with those of the professionals. Thus, identifying and understanding the factors associated with work-related stress and burnout, for example, allows for the development of personalized interventions that contribute to reducing illness and absenteeism, potentially even preventing professionals from leaving the field. In this light, moral resilience is introduced, as the understanding of its characteristics and their application to daily practice is believed to help plan strategies that assist healthcare professionals, particularly nurses, in responding to challenging situations.

Moral resilience is defined as the ability of an individual to sustain or restore their integrity in response to moral complexity, confusion, distress, or setbacks, and has been studied as a potential strategy for coping with moral distress ^(2,3)^. Studies ^(4-7)^ analyze its characteristics, which include personal or moral integrity, relational integrity, buoyancy, self-regulation, self-management, moral efficacy, and the qualities/skills of moral resilience.

Personal or moral integrity is understood as a “state of balance, harmony, and solidarity,” meaning remaining true to one’s values in the face of adversity ^(4-7)^. Relational integrity involves distinguishing one’s values from the perspectives and interests of others. Buoyancy refers to the capacity to “bounce back,” to be courageous when facing ethical challenges, and to resist threats to integrity, thus enhancing one’s ability to recover or preserve it, potentially gaining new perspectives while confronting challenges. Self-regulation involves being self-aware, flexible, and grounded, meaning the ability to remain authentic amid moral complexity. Self-management, meanwhile, refers to legitimizing and paying sufficient attention to one’s well-being, as well as recognizing one’s needs and limitations. Being able to care for and preserve integrity is necessary for an individual to exercise moral judgment and face ethically complex or distressing situations ^(5)^.

Moral efficacy is related to the importance of having the ability to advocate for what you believe is the right course of action or statement, even when faced with resistance ^(5)^. Finally, the qualities/skills necessary to promote moral resilience include the development of self-awareness and the capacity for introspection, investigation, active listening, and the use of reflective practices ^(5)^. Moral resilience is linked to awareness and self-awareness, respect for and appreciation of moral life, all of which play a fundamental role in its understanding ^(6)^.

Given the above, investing in moral resilience within oncology care becomes relevant, considering that healthcare professionals, especially nurses, face crises and ethical conflicts daily and need to develop coping strategies.

However, this reality has been scarcely explored in the literature, as only one study on moral resilience was found in Brazil between 2021 and 2024, which is believed to be an insufficient sample of specific studies on strategies to cultivate moral resilience in this country. Existing studies are mostly focused on healthcare professionals in adult and pediatric intensive care units, nurses and professionals working with COVID-19, and undergraduate nursing students, highlighting a knowledge gap regarding studies aimed at nurses working in hemato-oncology services. Research on moral resilience involving these professionals was not found. Based on this, the following research question is proposed: “What strategies do nurses in a hemato-oncology service use to develop moral resilience?”

## OBJECTIVE

To describe the strategies used by nurses in a hemato-oncology service for developing moral resilience.

## METHODS

### Ethical Aspects

This research adhered to Resolution No. 466/12 of the National Health Council of Brazil, and the project was reviewed and approved by the Research Ethics Committee. Each participant signed the Informed Consent Form (ICF), and a confidentiality agreement was attached to the project’s documents.

### Study Type

This is a qualitative, exploratory-descriptive study, presented according to the criteria of the Consolidated criteria for reporting qualitative research - COREQ checklist ^(8)^.

### Study Setting

The research was conducted in a public hospital in Rio Grande do Sul, Brazil, specifically within the hemato-oncology service. This service comprises five sectors: the Bone Marrow Transplant Service, Medical Clinic, Chemotherapy Outpatient Clinic, Pediatric Oncology Service, and Radiotherapy Service.

### Data Source

The population of these five sectors consists of a total of 50 nurses (16 from the Bone Marrow Transplant Service, 10 from the Medical Clinic, 12 from the Chemotherapy Outpatient Clinic, 10 from the Pediatric Oncology Service, and 2 from the Radiotherapy Service). Nurses with more than six months of experience in the sector were included, considering their adaptation time to the work process and their extended work experience. Nurses on vacation or on any type of leave during the data collection period were excluded.

### Data Collection and Organization

Data were collected from January to May 2023 through semi-structured interviews conducted by the study’s author. Prior to the study’s commencement, the author presented the research to the hemato-oncology services, inviting professionals to participate. Following the invitation, interview appointments were scheduled with nurses who were interested in participating. The interviews were conducted within the services, in a room provided by the nurses of each sector, with only the study’s author and each participant individually, ensuring privacy during data collection. The interviews began after the nurses signed the informed consent form, were recorded on a digital recorder, and lasted an average of 30 minutes. The nurses were selected using snowball sampling, with the first participant chosen being the reference nurse of the service.

The interview script included questions regarding the work process in the hemato-oncology unit, relationships with cancer patients and their families, interactions with the nursing and multidisciplinary teams, ethical practice, workplace conflicts, moral distress, challenges and ethical conflicts, perception of behaviors, coping strategies, management of ethical issues, and moral resilience. Example questions included: “What is your work process in the unit?”; “Do you identify conflicts in your workplace? Which ones?”; “How do you cope with or manage these issues?” The interviews were subsequently transcribed in full and returned to the participants for corrections or additional comments, thereby validating them for analysis. Data collection concluded when the study’s objectives were achieved.

### Data Analysis

Content analysis was used to analyze the data, performed in three phases: (1) pre-analysis, during which the research corpus was organized; (2) exploration of the material, involving a deeper examination of the content and the establishment of meaning units; and (3) inference and interpretation, involving the establishment of two thematic axes based on the approximation and frequency of statements, thereby addressing the study’s objective (9). The material was reviewed, relationships among the meaning units were examined, categories were constructed, and interpretations were made. The letter “R” was used to cite respondents, followed by the corresponding number according to their interview order.

## RESULTS

The research participants consisted of 14 nurses, of whom four were from the Bone Marrow Transplant Service, two from the Clinical Medical Unit, four from the Chemotherapy Outpatient Clinic, two from the Pediatric Oncology Unit, and two from the Radiotherapy Service. The majority of the nurses were female (n=13), aged between 30 and 50 years (n=14). They held either a lato sensu (n=09) or stricto sensu (n=05) postgraduate degree, and all had more than six months of experience at the institution (n=14).

From the analysis, two categories emerged: the first titled “Strategies at the Personal/Self-Awareness Level,” and the second, “Care Strategies/Patient Care.”

### Category 1: Strategies at the Personal/Self-Awareness Level

In this category, the nurses presented aspects related to coping with ethical conflicts in the workplace. According to these professionals, this coping process is complex, and moral resilience emerges as an individual strategy for personal care in situations where they recognize their limits, engage in external activities for self-care, and participate in team meetings. Insufficient psychological support was highlighted as a weakness of the institution, which can impact moral resilience.

[...] *Coping is difficult; you end up feeling this anguish and suffering. We don’t have support here-psychological, psychiatric, or otherwise. You try to cope in the best way you can, from a psychological perspective, so to speak, but we lack support. We try to resolve things, we reflect on them, but, undoubtedly, there will come a time when the suffering will be so overwhelming that you might fall into an abyss and not know how to escape.* (R2)

Nurses in hemato-oncology reported that, over time, they adapt to situations of suffering involving patients and families, which may or may not be linked to emotional distancing. It appears that daily experiences foster the development of a defensive and self-protective stance as a form of personal care, even though they do not consider this to be the most appropriate way to act.

[...] *When I first started here, it caused me a lot of suffering, but I think over time we get used to it. I’m not sure if that’s the right approach, but it also helps us avoid suffering too much because if we were to grieve for every patient we attend to, tomorrow we could be the ones in this situation. We could be the ones who are sick and experiencing psychological and physical suffering because it often happens. There are days that are quite overwhelming, especially with heavy cases, particularly involving children. It’s not an easy area to work in, and there are many family issues to consider.* (R9)

Learning to navigate the different perceptions of the team in response to the same situation was regarded by the nurses as a strategy for developing moral resilience, as respecting diverse opinions is essential for the effective functioning and collaboration of the team in patient care. Personal care strategies mentioned included therapy, engaging in physical activities, participating in research groups, exchanging experiences with other professionals, and understanding their competencies and limits of practice.

[...] *When I first started here, it was quite overwhelming, but I learned to respect the process as well. If something is done correctly, it will lead to the same outcome, so there’s no reason for me to intervene. However, if I see something that isn’t being done right and could harm the patient, I always try to find a way to address it, but never in front of the person or the patient.* (R11)

[...] *I feel much more resilient now, having a better understanding of my limits-knowing how far I can go and what is beyond my competence. Through the therapy I receive and my experience, along with studying and participating in research groups and discussion circles, stepping outside of this world of assistance and into the academic environment has helped me get to know myself better, understand the system, and find ways to protect myself and be resilient when I need to be.* (R6)

For some nurses, holding more frequent meetings with the team would be a way to strengthen moral resilience, as this would allow for reflection and the sharing of situations that require discussion, support, and assistance from the team. The theme of worker health is increasingly relevant, and workers are becoming more susceptible to illness.

[...] *One solution would be for the team to meet regularly to discuss service issues, and this also contributes to moral resilience. What we need to do-and what we can also achieve-is to support one another more and gain strength for our practice.* (R3)

[...] *The processes, dynamics, and issues surrounding the socialization of problems could be managed much better if there were better listening among colleagues and professionals. It’s not just about nurses; there’s a need to listen to custodians as well. It’s essential to truly hear and understand what people are going through. The technical aspects are important, but the dynamics often get overlooked in the workplace, especially regarding conflict management. There is pressure related to technical issues, but I believe that being aware of whether someone is suffering is crucial in today’s context, especially after coming out of a pandemic where professionals experienced significant distress, with many exhibiting symptoms or developing illnesses that are now, for example, forgotten.* (R8)

As previously mentioned, personal activities carried out outside of work hours contribute to the development of moral resilience. Clarity regarding competencies, responsibilities, and management by the nurse allows for better handling and coping with conflicting situations. Understanding the dynamics of multiprofessional teamwork and the limits of practice, according to their competencies, is crucial for nurses to recognize that, even when giving their best, there are issues that extend beyond their scope and should be addressed collaboratively. This understanding seems to alleviate the burden and suffering that professionals impose on themselves. However, these situations provoke moral distress, leading to feelings of anguish and helplessness as they realize they cannot manage them adequately.

[...] *I cope by seeing a therapist, exercising, taking care of myself, and taking time to reflect: this is mine, this is not mine; I cannot resolve this. I always give my best when caring for a patient, providing whatever they need-sometimes it’s a conversation or a referral to a nutritionist or psychologist, or requesting a clinical intervention for pain relief. That’s how I try to protect myself. Within what I can do, I do my best. This is mine, this is not mine; this is my responsibility, and that is not. However, we don’t always have this clarity. It’s when we lack clarity that we become overwhelmed and end up suffering.* (R6)

[...] *When there’s a terminal patient, sometimes the attitudes diverge. Last week, we had the death of a three-year-old child. Seeing that situation causes anguish, and you want to resolve it in another way, but sometimes you can’t because something prevents you from doing so. There’s the part I fulfill as a nurse, and there’s a part that is not mine, which I cannot handle or act upon. So, I depend on another professional to take a different approach. In situations like that, it’s quite difficult.* (R13)

### Category 2: Care Strategies/Patient Care

In this category, the nurses in hemato-oncology presented issues related to the perception of moral resilience in daily care practice. Care is integral to the professionals’ routine, but they do not always succeed in delivering what they believe to be best for the patient.

For the nurses, moral resilience is both a form of personal care and a means of caring for the patient, expressed through their advocacy for patient rights and intervention with the multidisciplinary team through effective communication.

[...] *There are various ways to advocate for the patient, and being resilient also means advocating for the patient. At that moment, I step back, consider what I can do, and then I engage-I sit down, talk, and advocate for the patient, but in a polite manner* [laughs]*, coherently, you know? Without exhausting myself and without exhausting my colleague as well. So, I am succeeding. It is indeed possible for us to be resilient in the work process.* (R6)

Some professionals struggle to provide care as they would like because they identify inconsistencies in the work process that contradict their beliefs and scientific knowledge, leading to moral distress. Nevertheless, they report that the primary focus and objective of their actions is always to prioritize the patient.

[...] *There are moments when you realize that you do not agree with a particular course of action, but since you are the one carrying out that order, so to speak, it gives the impression that you are doing something wrong. And if you don’t follow through, that’s also a problem, right?* (R3)

[...] *Our main objective is the patient. The patient encompasses all the issues I have with others concerning them. It’s not about my personal life.* (R4)

It was also observed that moral resilience is associated with respecting patient autonomy and striving for quality of life. In hemato-oncology, situations involving life and death are frequent, and although the nurses follow established protocols, they often hold differing opinions regarding the procedures to be performed. These professionals understand that it is their role to allow the patient to choose what will provide them with the greatest comfort and benefit, as in the case of palliative care versus therapeutic obstinacy. However, a conflict arises when, in the nurses’ view, there is insufficient clarification from the medical team regarding all invasive measures.

[...] *The issue of palliative care is very challenging; sometimes it takes a long time to decide that a patient will be placed in palliative care-not terminal care, but palliative care. For quite some time, we can see that the patient is palliative and has no prospect of a cure; however, the patient is not aware of this, which is something they should be the first to know, respecting their autonomy. They don’t know, the family doesn’t know, and we don’t provide the opportunity for the patient to have a good quality of life and to do what they want at the end of their life. We also do not prevent them from undergoing numerous procedures that will not lead to a cure. I believe this is a conflict for me. For a long time, doctors have known that the patient is palliative, yet they continue to insist on new measures and protocols, knowing that there is no prospect for a cure. They don’t ask whether the patient wants to continue with invasive procedures. Then, when the patient is already terminal, they call the family in and have a conversation with them, dropping that bombshell on both the family and the patient.* (R7)

Moral resilience was also identified in the ability to analyze situations, reflect, consider alternative paths, change approaches, step back, and continually rethink the best course of action.

[...] *In practice, even when you know what you need to do, you tell yourself, ‘I’m going to do this,’ but then you realize that it’s not feasible, that it won’t be effective, and you can’t accomplish what you’d like to do. At that point, you have to take a step back, reassess, and think again. Well, that didn’t work; let’s see how it goes. Return, rethink; if it didn’t work, it didn’t work.* (R8)

Some professionals stated that patient care is often hindered by hospital management issues, such as bed allocation and staffing levels, which they believe interferes with the development of moral resilience.

[...] *The institution is more concerned with numbers-quantity, bed occupancy, nursing staffing ratios, and so forth-than with quality.* [...] *Providing this service isn’t just about the numbers; it’s not just about looking at the map and seeing the number of available beds; there’s a whole process behind it. The institution isn’t focused on quality; it’s focused on quantity and numbers.* (R3)

## DISCUSSION

From the analysis, strategies at the personal and self-awareness levels were highlighted, as well as those employed in direct patient care and assistance management. In the first category, it was determined that, according to the nurses in hemato-oncology, developing individual strategies for personal care is essential to confront ethical conflicts in the workplace. Nursing is inherently linked to caring for the health of individuals; however, this care also leads to physical and emotional strain for professionals due to frequent exposure to stressors characteristic of the profession itself. In their daily practice, nurses must not only care for patients but also mediate conflicts, manage activities, and work as a team, which increases stress and contributes to the deterioration of their physical and mental health ^(10)^.

Nursing professionals are among those who experience the highest rates of illness due to occupational stress, which includes physiological and psychological reactions, mental disorders, work-related accidents, absences, and even high suicide rates ^(11)^. Factors such as pressure to meet targets, administrative issues, interpersonal relationships at work, task fragmentation, low autonomy, conflicts with management, competitiveness within their own ranks, job insecurity, excessive workloads, and unhealthy work environments are potential stressors for these professionals ^(12)^.

Additionally, it was observed that hemato-oncology nurses reported adapting over time to situations of suffering involving patients and families, which may be accompanied by emotional distancing. Self-protection is also a form of personal care and moral resilience. Studies demonstrate that the unpreparedness of professionals to deal with death and its consequences leads many to utilize distancing as a mechanism to minimize suffering. This creates a false sense of detachment from the patient and their illness, improving the psychological and emotional state of the team, thereby preventing interference with the execution of their professional activities ^(13)^.

In this research, hemato-oncology nurses identified therapy, engaging in physical activities, participating in research groups, exchanging experiences with other professionals, and understanding their competencies and limits of practice as forms of personal care. Strategies for coping with stress were outlined that closely align with those mentioned by nurses to promote moral resilience. One study ^(14)^ indicated that among the coping strategies for stress listed by nursing professionals at a university hospital, there were practices aimed at caring for both body and mind (recreation, physical activity, relaxation, integrative therapies), dialogue/support from family, friends, colleagues, and supervisors, distancing from problems, self-control, among others.

The nurses also emphasized that meetings with the team can be considered a way to develop moral resilience, as they create spaces for reflection, support, and assistance. Studies show that creating collective environments in healthcare institutions, where managers and professionals can express and discuss their ideas and opinions, legitimizes the experiences of these workers ^(15)^. Understanding that even when giving their best, there are situations beyond their control alleviates the burden that professionals impose on themselves.

However, in the second category, “Care Strategies/Patient Care,” it became evident that for the nurses, moral resilience is a form of personal care as well as a means of caring for the patient. Nevertheless, professionals do not always succeed in performing their activities as they would like and encounter discrepancies that contradict their beliefs and scientific knowledge. Such situations can lead to moral distress, which occurs when individuals feel unable to act according to what they believe is the appropriate course of action due to perceived constraints ^(16)^. Another study highlights that the lack of support for the nursing team causes dissatisfaction and insecurity in the workplace. Furthermore, the absence of support from colleagues, management, and the institution generates anxiety and even the desire to abandon the profession ^(17)^.

Another important factor is the perception of autonomy present in this care strategy, both concerning the patient and the nurse. Professional satisfaction is related to the ability of professionals to intervene in strategies that promote engagement and decision-making. This fosters greater involvement and commitment to the patient ^(18)^. Additionally, it is essential in caregiving practice to have communication directed toward the needs and expectations of patients, respecting their autonomy and preferences while using clear and accessible language ^(19)^.

Moral resilience also enables professionals to analyze situations, reflect, consider alternative paths, change approaches, step back, and continually rethink the best course of action. This aligns with the model of the total conscious spectrum structure, which suggests that reflection leads to an alignment of purposes essential for developing moral resilience ^(20)^.

### Limitations of the Study

A significant limitation of this study is that it was conducted in only one institution. Including other settings could provide a broader perspective on moral resilience. Therefore, similar research in other contexts involving hemato-oncology nurses is recommended.

### Contributions to Nursing, Health, or Public Policy

Our study makes significant contributions to the field of nursing by highlighting the strategies utilized by nurses in a hemato-oncology service to develop moral resilience. Moral resilience is a concept still underexplored in the national context, which also represents an important contribution to the nursing field. It is believed that discussions and focus on this topic may motivate nurses to confront challenging and conflicting situations, as well as promote reflections and potentially changes in the work process over time. Productions focused on worker health also enable the review of practices, and care increasingly demands critical thinking from nurses.

## FINAL CONSIDERATIONS

With this research, it was possible to describe the strategies employed by nurses in a hemato-oncology service for developing moral resilience. It was observed that, for hemato-oncology nurses, developing moral resilience strategies is related to what these professionals consider relevant, thinking not only of the patient but also of themselves.

Ethical conflicts in the workplace require professionals to adopt an ethical stance in the face of adversity. However, these professionals must also take care of themselves. There is a need for discussion and reflection spaces in the work environment, as this aids in planning care. Moral resilience also lies in the nurse’s ability to critically analyze each situation, seeking alignment of their objectives.

## References

[1] Garcia LG, Pinto MH, Canille RMDS. (2021). Engajamento do profissional da enfermagem no trabalho com crianças em tratamento oncológico. Enferm Foco.

[2] Rushton CH. (2016). Moral resilience: a capacity for navigating moral distress in critical care. AACN Adv Crit Care.

[3] Wocial LD. (2020). Resilience as an incomplete strategy for coping with moral distress in critical care nurses. Crit Care Nurse.

[4] Helmers A, Palmer KD, Greenberg RA. (2020). Moral distress: developing strategies from experience. Nurs Ethics.

[5] Holtz H, Heinze K, Rushton C. (2018). Interprofessionals’ definitions of moral resilience. J Clin Nurs.

[6] Defilippis TMLS, Curtis K, Gallagher A. (2020). Moral resilience through harmonised connectedness in intensive care nursing: a grounded theory study. Intensive Crit Care Nurs.

[7] Heinze KE, Hanson G, Holtz H, Swoboda SM, Rushton CH. (2021). Measuring health care interprofessionals’ moral resilience: validation of the Rushton Moral Resilience Scale. J Palliat Med.

[8] Souza VRDS, Marziale MHP, Silva GTR, Nascimento PL. (2021). Tradução e validação para a língua portuguesa e avaliação do guia COREQ. Acta Paul Enferm.

[9] Bardin L. (2016). Análise de conteúdo.

[10] Reis CD, Amestoy SC, Silva GTRD, Santos SDD, Varanda PAG, Santos IARD (2020). Situações estressoras e estratégias de enfrentamento adotadas por enfermeiras líderes. Acta Paul Enferm.

[11] Bardaquim VA, Santos SVMD, Dias EG, Dalri RDCDMB, Mendes AMDOC, Gallani MC (2020). Stress and cortisol levels among members of the nursing team. Rev Bras Enferm.

[12] Miranda ARDO, Afonso MLM. (2021). Occupational stress in nurses: a critical view in times of pandemics. Braz J Develop.

[13] Salimena AMDO, Teixeira SDR, Amorim TV, Paiva ADCPC, Melo MCSCD. (2013). Estratégias de enfrentamento usadas por enfermeiros ao cuidar de pacientes oncológicos. Rev Enferm UFSM.

[14] Baccin AA, Lucchese VC, Lima ALDD, Medeiros BVDS, Maliska JKDL, Vasconcellos SJL. (2023). Estratégias de enfrentamento ao estresse e engajamento no trabalho da equipe de enfermagem hospitalar. Rev Eletrôn Acervo Saúde.

[15] Oliveira AFDC, Teixeira ER, Athanázio AR, Soares RDS. (2021). Sofrimento psíquico e a psicodinâmica no ambiente de trabalho do enfermeiro: revisão integrativa. Online Braz J Nurs.

[16] Jameton A. (1984). Nursing practice: the ethical issues.

[17] Maben J, Bridges J. (2020). COVID-19: Supporting nurses’ psychological and mental health. J Clin Nurs.

[18] Dias S, Morais C. (2020). Satisfação e engagement: (re)pensar a saúde e o bem-estar dos enfermeiros. Rev Port Enferm Saúde Mental.

[19] Andrade KGM. (2022). Cuidados paliativos de enfermagem ao paciente oncológico em estado terminal. Rev Interdisc Pens Científ.

[20] Rushton CH (2018). Moral Resilience.

