# Supplementary material for: Strategies for the development of moral resilience: voices of oncology nurses
Source: Rev Bras Enferm. 2025 Mar 14;78(2):e20240210. doi: 10.1590/0034-7167-2024-0210 (PMC11913130; doi:10.1590/0034-7167-2024-0210)
Supplement: Translation

# Translation: Estratégias para o desenvolvimento da resiliência moral: vozes de enfermeiros da oncologia

**DOI:** 10.1590/0034-7167-2024-0210pt

**Authors:** Mariane da Silva Barbosa, Ariel Siqueira Lemos, Liliane Alves Pereira, Taís Carpes Lanes, Flávia de Mello Disconsi, Kellen da Costa Pompeu, Jordana Lopes Carvalho, Graziele de Lima Dalmolin

**Affiliations:** I Universidade Federal de Santa Maria. Santa Maria, Rio Grande do Sul, Brasil; II Universidade Federal de Santa Maria, Hospital Universitário. Santa Maria, Rio Grande do Sul, Brasil; III Universidade Federal do Rio Grande. Rio Grande, Rio Grande do Sul, Brasil; IV Centro Universitário Franciscano. Santa Maria, Rio Grande do Sul, Brasil

**Keywords:** Oncologia, Resiliência Psicológica, Enfermagem, Ética, Moral

## Abstract

**Objetivo:**

descrever as estratégias utilizadas pelos enfermeiros de um serviço de hemato-oncologia para o desenvolvimento da resiliência moral.

**Métodos:**

pesquisa qualitativa, exploratório-descritiva, realizada com enfermeiros do serviço de hemato-oncologia de uma instituição hospitalar pública do Rio Grande do Sul. A coleta de dados ocorreu no período de janeiro a maio de 2023, por meio de entrevistas semiestruturadas. Empregou-se análise de conteúdo.

**Resultados:**

participaram do estudo 14 enfermeiros. As questões levantadas sobre resiliência moral resultaram em duas categorias. A primeira sendo estratégias a nível pessoal/autoconhecimento e, a segunda, estratégias de cuidados/cuidados com o paciente.

**Considerações finais:**

observou-se que para os enfermeiros da hemato-oncologia, desenvolver estratégias de resiliência moral está relacionado ao que esses profissionais acreditam ser relevante pensando não somente no paciente, mas também em si próprios.

## INTRODUÇÃO

Com o envelhecimento populacional e o advento das doenças crônicas não transmissíveis, dentre elas o câncer, exige-se dos profissionais de saúde preparo técnico e científico para o cuidado qualificado e seguro a esses pacientes. A confirmação de um diagnóstico de câncer, em diversos momentos, é associada à morte, o que torna difícil, para os profissionais envolvidos no cuidado, a abordagem e o enfrentamento desse contexto ^(1)^.

Diante disso, torna-se imperativo que esses profissionais tenham a possibilidade de enfrentamento do estresse, burnout, desgaste e sofrimento moral, visto que, em muitos momentos, a situação de doença do paciente transcende o cuidado ou envolve valores que não coadunam com os dos profissionais. Assim, a identificação e o entendimento dos fatores associados ao estresse relacionado ao trabalho e à síndrome de burnout, por exemplo, permitem que sejam desenvolvidas intervenções personalizadas, que contribuem para a redução do adoecimento e afastamentos do trabalho, podendo até mesmo evitar o abandono da profissão pelos profissionais. Nesse sentido, insere-se a resiliência moral, uma vez que, com a compreensão de algumas de suas características e, associando-as à prática diária, acredita-se que seja possível planejar estratégias que auxiliem na resposta dos profissionais, em especial dos enfermeiros, diante de situações desafiadoras.

Resiliência moral é definida como a capacidade de um indivíduo de sustentar ou restaurar sua integridade em resposta à complexidade moral, confusão, angústia ou contratempos, e tem sido estudada como possível estratégia para o enfrentamento do sofrimento moral ^(2,3)^. Estudos ^(4-7)^ analisam suas características, dentre as quais se citam: integridade pessoal ou moral; integridade relacional; flutuação; autorregulação; autogestão; eficácia moral e qualidades/habilidades de resiliência moral.

A integridade pessoal ou moral é entendida como “estado de equilíbrio, harmonia e solidariedade”, ou seja, permanecer fiel aos próprios valores em meio à adversidade ^(4-7)^. A integridade relacional busca distinguir os próprios valores dos pontos de vista e interesses dos outros. Flutuação seria a capacidade de “se recuperar”, ser corajoso ao enfrentar desafios éticos e resistir a ameaças à integridade, potencializando suas capacidades para recuperá-la ou preservá-la, podendo adquirir novas perspectivas enquanto enfrenta os desafios. A autorregulação envolve ser autoconsciente, flexível e fundamentado, ou seja, a capacidade de permanecer autêntico em meio à complexidade moral. Já a autogestão refere-se a legitimar e dar atenção suficiente ao bem-estar, bem como reconhecer suas necessidades e limitações. Ser capaz de cuidar e preservar a integridade é necessário para que o indivíduo exerça o arbítrio moral e enfrente situações eticamente complexas ou angustiantes ^(5)^.

A eficácia moral está relacionada à importância de ter a capacidade de defender o que você acredita ser a coisa certa a fazer ou dizer, mesmo quando se depara com resistência ^(5)^. E, por fim, as qualidades/habilidades para promover a resiliência moral incluem o desenvolvimento da autoconsciência e a capacidade de introspecção, investigação, exercício de escuta e uso de práticas reflexivas ^(5)^. A resiliência moral se relaciona com a consciência e autoconsciência, respeito e apreço à vida moral, as quais possuem papel fundamental na sua compreensão ^(6)^.

Diante do exposto, o investimento na resiliência moral no contexto do cuidado em oncologia torna-se relevante, considerando que os profissionais de saúde, em especial os enfermeiros, lidam diariamente com situações de crise e conflitos éticos e necessitam desenvolver estratégias de enfrentamento.

No entanto, essa realidade tem sido pouco explorada na literatura, visto que no Brasil, no período de 2021 a 2024, foi encontrado apenas um estudo sobre a resiliência moral, o que se acredita ser uma amostra insuficiente de estudos específicos sobre estratégias para cultivar a resiliência moral nesse país. Os estudos existentes são, em sua maioria, com profissionais de saúde de unidades de terapia intensiva adultas e pediátricas, enfermeiros e profissionais atuantes na COVID-19 e com estudantes de graduação em enfermagem, o que demonstra uma lacuna de conhecimento para estudos voltados a enfermeiros que atuam em serviços de hemato-oncologia, uma vez que pesquisas com a temática de resiliência moral não foram encontradas com esses profissionais. Com base no exposto, propõe-se a seguinte questão de pesquisa: “Quais estratégias os enfermeiros de um serviço de hemato-oncologia utilizam para o desenvolvimento da resiliência moral?”.

## OBJETIVO

Descrever as estratégias utilizadas pelos enfermeiros de um serviço de hemato-oncologia para o desenvolvimento da resiliência moral.

## MÉTODOS

### Aspectos éticos

A presente pesquisa respeitou a Resolução nº 466/12 do Conselho Nacional de Saúde do Brasil, e o projeto foi avaliado e aprovado pelo Comitê de Ética em Pesquisa. Cada participante assinou o Termo de Consentimento Livre e Esclarecido (TCLE), e o termo de confidencialidade foi vinculado aos documentos do projeto.

### Tipo de estudo

Trata-se de uma pesquisa qualitativa, do tipo exploratório-descritiva, apresentada segundo os critérios do checklist *Consolidated criteria for reporting qualitative research* -COREQ ^(8)^.

### Cenário do estudo

A pesquisa foi realizada em uma instituição hospitalar pública do Rio Grande do Sul, especificamente no serviço de hemato-oncologia. O serviço é composto por cinco setores, sendo eles: Serviço de Transplante de Medula Óssea, Clínica Médica, Ambulatório de Quimioterapia, Serviço de Tratamento da Criança com Câncer e Serviço de Radioterapia.

### Fonte de dados

A população desses cinco setores compreende um total de 50 enfermeiros (16 enfermeiros do Serviço de Transplante de Medula Óssea, 10 do Serviço de Clínica Médica, 12 do Ambulatório de Quimioterapia, 10 do Serviço de Tratamento da Criança com Câncer e 2 do Serviço de Radioterapia). Foram incluídos enfermeiros com mais de seis meses de trabalho no setor, considerando seu tempo de adaptação ao processo de trabalho e maior período de vivência. Foram excluídos enfermeiros em férias ou em licença de qualquer natureza no período de coleta de dados.

### Coleta e organização dos dados

A coleta de dados ocorreu de janeiro a maio de 2023, por meio de entrevistas semiestruturadas, realizadas pela autora do estudo. Antes do início da pesquisa, a autora apresentou o estudo aos serviços de hemato-oncologia, convidando os profissionais a participarem. Após o convite, foi realizado o agendamento das entrevistas com os enfermeiros interessados em participar do estudo. As entrevistas foram realizadas nos serviços, em uma sala disponibilizada pelos enfermeiros de cada setor, somente com a autora do estudo e os participantes individualmente, garantindo a privacidade para a coleta de dados. As entrevistas foram iniciadas após a assinatura do termo de consentimento pelos profissionais, registradas em um gravador digital, com tempo médio de duração de 30 minutos. Os enfermeiros foram selecionados por amostragem bola de neve, sendo que o primeiro participante selecionado foi o enfermeiro referência do serviço.

O roteiro das entrevistas foi composto por questionamentos envolvendo o processo de trabalho na unidade hemato-oncológica, relação com o paciente oncológico e sua família, relação com a equipe de enfermagem e multiprofissional, prática ética, conflitos no ambiente de trabalho, sofrimento moral, desafios e conflitos éticos, percepção de condutas, formas de enfrentamento, gerenciamento de questões éticas e resiliência moral. Exemplos de perguntas: “Como é o seu processo de trabalho na unidade?” “Você identifica situações de conflito no seu ambiente de trabalho? Quais?” “Como você enfrenta/gerencia essas questões?” As entrevistas foram posteriormente transcritas na íntegra, sendo devolvidas aos participantes para correção ou inclusão de comentários, validando-as para análise. A etapa de coleta de dados foi finalizada quando os objetivos do estudo foram alcançados.

### Análise dos dados

Para a análise dos dados, utilizou-se a análise de conteúdo, realizada em três fases: (1) pré-análise, quando o corpus da pesquisa foi organizado; (2) exploração do material, com o aprofundamento no material e o estabelecimento de unidades de sentido; (3) inferência e interpretação, com o estabelecimento dos dois eixos temáticos por aproximação e frequência das falas, respondendo ao objetivo do estudo ^(9)^. Foram realizadas leituras do material, aproximação e verificação de relações entre as unidades de sentido, construção das categorias e interpretação. A letra “R” foi utilizada para a citação dos respondentes, seguida do número correspondente ao indivíduo conforme sua ordem de entrevista.

## RESULTADOS

Os participantes da pesquisa foram 14 enfermeiros, dos quais quatro eram do Serviço de Transplante de Medula Óssea, dois da unidade de internação Clínica Médica, quatro do Ambulatório de Quimioterapia, dois da unidade de internação para crianças com câncer e dois do Serviço de Radioterapia. A maioria dos enfermeiros era do sexo feminino (n=13), com idades entre 30 e 50 anos (n=14). Eles possuíam pós-graduação em nível lato sensu (n=09) ou stricto sensu (n=05), com mais de seis meses de atuação na instituição (n=14).

A partir da análise, emergiram duas categorias: a primeira, denominada “Estratégias em nível pessoal/autoconhecimento”, e a segunda, “Estratégias de cuidados/cuidados com o paciente”.

### Categoria 1: Estratégias em nível pessoal/autoconhecimento

Nesta categoria, os enfermeiros apresentaram aspectos relacionados ao enfrentamento de conflitos éticos no ambiente de trabalho. De acordo com esses profissionais, esse enfrentamento é complexo, e a resiliência moral surge como uma estratégia individual de cuidado pessoal nas situações de reconhecimento de seus limites, realização de atividades externas para autocuidado e participação em reuniões com a equipe de trabalho. O apoio psicológico insuficiente foi destacado como uma fragilidade da instituição, o que pode impactar a resiliência moral.

[...] *O enfrentamento é difícil, acaba que tu fica nessa angústia, nesse sofrimento. A gente não tem um apoio aqui, apoio psicológico, psiquiátrico, enfim. Tu tenta enfrentar da forma que consegue, no teu aspecto psicológico, vamos dizer assim, mas a gente não tem um apoio. A gente tenta resolver as coisas, a gente reflete sobre isso, mas, com certeza, vai chegar uma hora que o sofrimento vai ser tanto que, talvez, tu caia em um abismo que não saiba como sair.* (R2)

Os enfermeiros da hemato-oncologia relataram que, com o tempo, ocorre uma adaptação às situações de sofrimento envolvendo pacientes e familiares, o que pode ou não estar ligado a um distanciamento emocional. Percebe-se, aparentemente, que o cotidiano provoca o desenvolvimento de uma postura de defesa e autoproteção como forma de cuidado pessoal, ainda que não considerem essa a maneira mais adequada de agir.

[...] *Logo quando eu entrei aqui, isso me trazia bastante sofrimento, mas acho que com o passar do tempo a gente vai acostumando. Não sei se é o certo, só que isso também faz com que a gente não sofra tanto, porque se a gente entrar em sofrimento por cada paciente que a gente vai atender, amanhã é a gente que está aqui. A gente que vai estar doente, vai entrar em sofrimento psíquico, físico, porque muitas vezes acontece, tem dias bem carregados, de pacientes pesados, crianças principalmente, não é uma parte fácil de trabalhar, tem toda a questão familiar.* (R9)

Aprender a lidar com as diferentes percepções da equipe diante de uma mesma situação foi considerado pelos enfermeiros uma estratégia para desenvolver a resiliência moral, visto que respeitar as diferentes opiniões é essencial para o bom funcionamento e a atuação da equipe no cuidado ao paciente. Também foram mencionados como cuidados pessoais a terapia, a prática de atividades físicas, a participação em grupos de pesquisa, a troca de experiências com outros profissionais, bem como a compreensão de suas competências e limites de atuação.

[...] *Mas aprendi a respeitar também. Se aquilo ali está certo, vai dar no mesmo caminho, não tem porque me meter. Agora, se, por exemplo, tu vê que tem alguma coisa, não é assim que se faz, isso aí vai prejudicar o paciente, aí sempre tento dar um jeitinho, mas nunca na frente da pessoa, nem na frente do paciente.* (R11)

[...] *Estou bem mais resiliente, tendo essa compreensão dos meus limites, até onde posso ir e onde não é da minha competência. Mas, com a terapia que faço e com a experiência, até essa questão de estudo, de participar do grupo de pesquisa, dessas rodas de conversa, sair aqui desse mundo, sair da assistência, ir para a academia, isso também fez eu me conhecer, conhecer o sistema e as formas de me proteger, ser resiliente quando precisa ser resiliente.* (R6)

Para outros enfermeiros, a realização de reuniões mais frequentes com a equipe seria uma forma de fortalecer a resiliência moral, pois isso permitiria a reflexão e o compartilhamento de situações que exigem discussão, apoio e suporte da equipe. A temática da saúde do trabalhador é cada vez mais atual, e os trabalhadores estão se tornando cada vez mais adoecidos.

[...] *Então uma solução seria a equipe se reunir regularmente para discutir as questões do serviço e isso já vem com a resiliência moral também. O que a gente tem que fazer e a gente consegue também é se apoiar mais um no outro e adquirir mais força, para a nossa prática.* (R3)

[...] *Os processos, as dinâmicas, as questões de socialização dos problemas, elas poderiam ser muito melhor administradas se houvesse uma escuta melhor dos próprios colegas, dos profissionais, não só falando de profissionais, só de enfermeiros, existe a necessidade de escutar o faxineiro também, existe a necessidade de tu conseguir escutar mesmo, entender o que está se passando com as pessoas. A parte técnica é a parte técnica, a parte das dinâmicas são muito esquecidas nos ambientes de trabalho, inclusive para gerenciar conflito, não há. Existe a cobrança da questão técnica. Acho que essa preocupação de saber se tem alguém em sofrimento é imprescindível nos dias atuais, que a gente acabou saindo de uma pandemia onde os profissionais sofreram muito, muitos tiveram sintomas ou desenvolveram também doenças e que, hoje, por exemplo, são esquecida.* (R8)

Como já mencionado, atividades de cunho pessoal realizadas fora do horário de trabalho ajudam no desenvolvimento da resiliência moral. A clareza das competências, responsabilidades e gestão do enfermeiro permite um melhor gerenciamento, condução e enfrentamento de situações conflituosas. A compreensão do trabalho em equipe multiprofissional e dos limites de atuação, conforme suas competências, é fundamental para o enfermeiro entender que, mesmo dando o melhor de si, existem questões que ultrapassam seu âmbito de atuação e devem ser resolvidas em conjunto. Essa compreensão parece aliviar a sobrecarga e o sofrimento que o profissional atribui a si mesmo. No entanto, essas situações provocam sofrimento moral, com sentimentos de angústia e impotência ao perceberem que não conseguem lidar com elas adequadamente.

[...] *Enfrento fazendo terapia com psicólogo, fazendo exercício físico, cuidando de mim, tirando tempo para mim, sentando e pensando: isso é meu, isso não é meu, não vou ter como resolver. Fazendo o melhor de mim quando vou cuidar do paciente, sempre dando o melhor para aquele paciente, seja o que ele necessita, às vezes é a conversa ou é um encaminhamento para uma nutricionista, para um psicólogo, solicitando uma intervenção clínica para alívio da dor. É assim que tento me proteger, dentro do que posso fazer, faço o melhor. Isso é meu, isso não é meu, isso está sob a minha responsabilidade, isso não está. Mas, nem sempre a gente consegue ter essa clareza. No momento que a gente não tem clareza, é quando a gente se sobrecarrega, aí acaba sofrendo.* (R6)

[...] *Quando tem um paciente terminal, às vezes, diverge as atitudes. Semana passada a gente teve um óbito de uma criança de três anos. Então, tu vendo aquela situação, vai te angustiando, querendo resolver de uma outra forma e, às vezes, não pode, porque tem uma coisa que te impede. Tem a parte que eu faço como enfermeira e tem uma parte que não é minha, que eu não tenho como fazer, atuar. Então, dependo de outro profissional de ter aquela atitude diferenciada. E nessas situações, assim, é bem difícil.* (R13)

### Categoria 2: Estratégias de cuidados/cuidados com o paciente

Nesta categoria, os enfermeiros da hemato-oncologia apresentaram questões relacionadas à percepção da resiliência moral na prática assistencial diária. O cuidado está presente na rotina dos profissionais, mas nem sempre eles conseguem realizar o que consideram ser o melhor para o paciente.

Para os enfermeiros, a resiliência moral é uma forma de cuidado pessoal e também de cuidado com o paciente, expressando-se por meio da atuação na defesa dos direitos dos pacientes e da intervenção junto à equipe multiprofissional através de uma comunicação adequada.

[...] *Existem várias formas de brigar pelo paciente e, ser resiliente, também é brigar pelo paciente. Nesse momento, me afasto, penso no que posso fazer e aí sim vou sentar, vou conversar, brigar pelo paciente, mas de uma forma educada* [risos]*, coerente, sabe? Sem me desgastar e sem que desgaste o colega também. Então, estou sendo, estou conseguindo. E é possível, sim, a gente ser resiliente no processo de trabalho.* (R6)

Alguns profissionais nem sempre conseguem prestar o cuidado da forma que gostariam, pois identificam incoerências no processo de trabalho que vão contra suas crenças e conhecimentos científicos, o que pode gerar sofrimento moral. No entanto, relatam que o foco principal e objetivo das suas condutas é sempre pensar no paciente.

[...] *Tem momentos que tu percebe que não está de acordo com aquela conduta, mas como tu é o executor daquela ordem, entre aspas, te dá a impressão que tu está fazendo a coisa errada e daí se tu não faz é um problema também né.* (R3)

[...] *Nosso objetivo principal é o paciente. O paciente envolve todas as questões que tenho com os outros em relação a eles. Não é sobre a minha vida pessoal.* (R4)

Observou-se também que a resiliência moral está associada ao respeito à autonomia do paciente e à busca pela qualidade de vida. Em hemato-oncologia, situações envolvendo vida e morte são frequentes, e os enfermeiros, embora sigam os protocolos estabelecidos, muitas vezes têm opiniões diferentes em relação aos procedimentos a serem realizados. Esses profissionais entendem que é seu papel permitir que o paciente opte pelo que lhe trará maior conforto e benefício, como no caso de cuidados paliativos versus obstinação terapêutica. No entanto, há um conflito quando, na visão dos enfermeiros, não há esclarecimento suficiente por parte da equipe médica sobre todas as medidas invasivas.

[...] *A questão dos cuidados paliativos, é bem difícil, às vezes demoram muito para decidir que o paciente vai ser paliativo. Não terminal, paliativo. A gente há muito já vê que ele é paliativo, que ele não tem perspectiva de cura, só que daí ele não sabe, que era a primeira pessoa que deveria saber, o paciente, respeitar a autonomia dele. Ele não sabe, a família não sabe e a gente não oportuniza que ele tenha uma qualidade de vida e que ela faça o que ele quer no final da vida dele. E não proporciona também que ele não passe por tantos procedimentos que não vão levar a cura. Acho que isso é um conflito para mim. Há muito tempo os médicos sabem que ele é paliativo, mas ficam insistindo em novas medidas, novos protocolos, que eles sabem que não vai ter mais perspectiva de cura, não questionam se ele quer ou não continuar com procedimentos invasivos. Daí quando já está terminal que chama a família, conversa com a família, cai aquela bomba na família e no paciente.* (R7)

A resiliência moral também foi identificada na capacidade de analisar as situações, refletir, ponderar outros caminhos, mudar condutas, recuar e sempre repensar a melhor forma de atuação.

[...] *E na prática mesmo que muitas vezes tu sabe o que tem que fazer, tu se coloca “ai, eu vou fazer” e daqui a pouco percebe que não dá, que não é por aí, não vai ser efetivo, não é factível conseguir fazer o que gostaria de fazer, e aí tem que dar um passo atrás, voltar e repensar. Bom, não deu, vamos ver como é. Retornar, repensar, não deu, não deu.* (R8)

Na fala de alguns profissionais, o cuidado ao paciente muitas vezes é prejudicado por questões de gestão hospitalar, como a distribuição de leitos e o dimensionamento de profissionais, o que, na visão deles, interfere no desenvolvimento da resiliência moral.

[...] *A instituição está mais preocupada com números, quantidade, leitos, ocupação de leitos, dimensionamento de enfermagem e afins, pouco preocupada com a qualidade.* [...] *Para prestar esse serviço, então não é só numeruzinho, não é só o mapinha que tu abre lá vê a quantidade de leitos, tem todo um processo por trás. A instituição não está preocupada com qualidade, está preocupada com quantidade, com números.* (R3)

## DISCUSSÃO

A partir da análise, evidenciaram-se estratégias em nível pessoal e de autoconhecimento, bem como aquelas empregadas no cuidado direto aos pacientes e no gerenciamento da assistência. Na primeira categoria, identificou-se que, de acordo com os enfermeiros da hemato-oncologia, para enfrentar conflitos éticos no ambiente de trabalho, é preciso desenvolver estratégias individuais de cuidado pessoal. A enfermagem tem sua identidade relacionada ao cuidado com a saúde do indivíduo; no entanto, esse cuidado também gera desgaste físico e emocional para o profissional, devido à exposição frequente a fatores estressantes, característicos do próprio trabalho. No dia a dia da profissão, o enfermeiro, além de cuidar do paciente, precisa mediar conflitos, gerenciar atividades e trabalhar em equipe, o que aumenta o estresse no exercício da profissão e contribui para o desenvolvimento de agravos à sua saúde física e mental ^(10)^.

Os profissionais de enfermagem são os que mais adoecem devido ao estresse ocupacional, o que inclui reações fisiológicas e psicológicas, transtornos mentais, acidentes de trabalho, afastamentos e até mesmo altas taxas de suicídio ^(11)^. Fatores como a pressão por metas, questões administrativas, relacionamentos interpessoais no trabalho, fragmentação de tarefas, baixa autonomia, conflitos com a chefia, competitividade dentro da própria classe, insegurança em relação ao emprego, sobrecarga de serviço e ambientes insalubres são potenciais estressores para esses profissionais ^(12)^.

Ainda nessa categoria, observou-se que os enfermeiros da hemato-oncologia relataram que, com o tempo, ocorre adaptação diante das situações de sofrimento envolvendo pacientes e familiares, bem como um distanciamento emocional. A autoproteção também é uma forma de cuidado pessoal e de Resiliência Moral. Estudos demonstram que o despreparo dos profissionais para lidar com a morte e suas consequências faz com que muitos utilizem o afastamento como mecanismo para minimizar o sofrimento. Isso cria uma falsa sensação de distanciamento do paciente e de sua doença, melhorando o estado psicoemocional da equipe, para que isso não interfira no desenvolvimento de suas atividades profissionais ^(13)^.

Nesta pesquisa, os enfermeiros da hemato-oncologia especificaram a terapia, a prática de atividade física, a participação em grupos de pesquisa, a troca de experiências com outros profissionais e a compreensão de suas competências e limites de atuação como formas de cuidado pessoal. Foram elencadas estratégias para enfrentamento do estresse que se aproximam das estratégias elencadas pelos enfermeiros para promover a resiliência moral. Um estudo ^(14)^ apontou que, entre as estratégias de enfrentamento do estresse listadas por profissionais de enfermagem de um hospital universitário, estavam cuidados com o corpo e a mente (lazer, atividade física, relaxamento, terapias integrativas), diálogo/apoio de familiares, amigos, colegas e chefias, afastamento do problema, autocontrole, entre outras.

Os enfermeiros também destacaram que reuniões com a equipe podem ser consideradas uma forma de desenvolver Resiliência Moral, pois possibilitariam espaços para reflexões, apoio e suporte. Estudos demonstram que é relevante a criação de ambientes coletivos nas instituições de saúde onde gestores e profissionais possam expressar e discutir suas ideias e opiniões, legitimando assim a experiência desses trabalhadores ^(15)^. A compreensão de que, mesmo dando o melhor de si, existem situações que fogem ao seu controle, alivia a sobrecarga que o profissional impõe a si mesmo.

Contudo, na segunda categoria, “Estratégias de cuidados/cuidados com o paciente”, evidenciou-se que, para os enfermeiros, Resiliência Moral é uma forma de cuidado pessoal e também de cuidado com o paciente. Entretanto, os profissionais nem sempre conseguem desempenhar suas atividades como gostariam e encontram divergências que vão contra suas crenças e conhecimento científico. Esse tipo de situação pode ser fonte de sofrimento moral, que ocorre quando o indivíduo é incapaz de realizar o que acredita ser o curso de ação adequado, devido a restrições percebidas nessa ação ^(16)^. Outro estudo destaca que a falta de suporte para a equipe de enfermagem causa insatisfação e insegurança no ambiente de trabalho. Ademais, a falta de apoio de colegas, chefias e da instituição gera angústias e até mesmo o desejo de abandonar a profissão ^(17)^.

Outro fator importante é a percepção de autonomia presente nessa estratégia de cuidado, tanto em relação ao paciente quanto ao enfermeiro. A satisfação profissional está relacionada à capacidade do profissional de intervir em estratégias que promovam engajamento e tomada de decisões. Isso possibilita maior envolvimento e comprometimento com o paciente ^(18)^. Além disso, considera-se indispensável, na prática assistencial, uma comunicação voltada para as necessidades e expectativas dos pacientes, que respeite sua autonomia e preferências, utilizando uma linguagem clara e acessível ^(19)^.

Identifica-se também que a Resiliência Moral possibilita analisar as situações, refletir, ponderar sobre outros caminhos, mudar condutas, voltar atrás e repensar sempre a melhor forma de atuação, o que converge com o modelo da estrutura de espectro total consciente, que apresenta a ideia de que a reflexão leva a um alinhamento de propósitos, essencial para o desenvolvimento da resiliência moral ^(20)^.

### Limitações do Estudo

Como limitação, destaca-se a realização do estudo em apenas uma instituição. A inclusão de outros cenários poderia permitir uma percepção mais ampla da resiliência moral. Assim, sugere-se a realização de pesquisas semelhantes em outros contextos de atuação dos enfermeiros/as de hemato-oncologia.

### Contribuições para a área da Enfermagem, Saúde ou Política Pública

Nosso estudo contribui significativamente para o campo da Enfermagem ao evidenciar as estratégias utilizadas pelos enfermeiros de um serviço de hemato-oncologia para o desenvolvimento da resiliência moral. A resiliência moral é um conceito ainda pouco explorado no contexto nacional, o que também representa uma contribuição importante para a área da Enfermagem. Acredita-se que a discussão e o enfoque nessa temática possam motivar os enfermeiros a enfrentarem situações difíceis e conflituosas, além de promover reflexões e possivelmente mudanças no processo de trabalho ao longo do tempo. Produções com enfoque na saúde do trabalhador também possibilitam a revisão de condutas, e a assistência exige, cada vez mais, pensamento crítico dos enfermeiros.

## CONSIDERAÇÕES FINAIS

Com a presente pesquisa, foi possível descrever as estratégias utilizadas pelos enfermeiros de um serviço de hemato-oncologia para o desenvolvimento da resiliência moral. Observou-se que, para os enfermeiros da hemato-oncologia, desenvolver estratégias de Resiliência Moral está relacionado ao que esses profissionais acreditam ser relevante, pensando não apenas no paciente, mas também em si mesmos.

Conflitos éticos no ambiente de trabalho exigem que o profissional adote uma postura ética diante da adversidade. No entanto, esse profissional também deve assumir uma postura de cuidado consigo. Há necessidade de espaços de discussão e reflexão no ambiente de trabalho, uma vez que isso auxilia no planejamento do cuidado. A Resiliência Moral também está na capacidade do enfermeiro de analisar criticamente cada situação, buscando o alinhamento de seus objetivos.
